# Mlcoalsim: Multilocus Coalescent Simulations

**Published:** 2007-03-02

**Authors:** Sebastian E. Ramos-Onsins, Thomas Mitchell-Olds

**Affiliations:** 1 Max-Planck Institute for Chemical Ecology, Hans-Knöll Str. 8, D-07745 Jena, Germany; 2 Present address: Departament de Genètica, Universitat de Barcelona, Diagonal 645, Barcelona, Spain; 3 Present address: Department of Biology, Duke University, Durham, NC 27708, USA

**Keywords:** Neutrality tests, Rejection algorithm, Population Genetics, Multilocus analyses, Coalescent simulations

## Abstract

Coalescent theory is a powerful tool for population geneticists as well as molecular biologists interested in understanding the patterns and levels of DNA variation. Using coalescent Monte Carlo simulations it is possible to obtain the empirical distributions for a number of statistics across a wide range of evolutionary models; these distributions can be used to test evolutionary hypotheses using experimental data. The *mlcoalsim* application presented here (based on a version of the *ms* program, Hudson, 2002) adds important new features to improve methodology (uncertainty and conditional methods for mutation and recombination), models (including strong positive selection, finite sites and heterogeneity in mutation and recombination rates) and analyses (calculating a number of statistics used in population genetics and *P*-values for observed data). One of the most important features of *mlcoalsim* is the analysis of multilocus data in linked and independent regions. In summary, *mlcoalsim* is an integrated software application aimed at researchers interested in molecular evolution. *mlcoalsim* is written in ANSI C and is available at: http://www.ub.es/softevol/mlcoalsim.

## Introduction

Statistical inference of molecular population data under different evolutionary models typically employs a coalescent framework (Kingman, 1982a,b; Hudson, 1990; Donnelly and Tavaré, 1995; Nordborg, 2001). Hudson’s *ms* (Hudson, 2002) application enabled a large number of population geneticists and molecular biologists to examine data under different evolutionary models. In recent years, a number of coalescent programs focused on the generation of genetic data have been published (e.g. *SimCoal*, Excoffier et al. 2000; Laval and Excoffier, 2004; *SelSim*, Spencer and Coop, 2004; *CoaSim*, Mailund et al 2005; *FastCoal*, Marjoram and Wall, 2005). Nevertheless, multilocus data obtained by high throughput techniques (e.g. the Drosophila Polymorphisms Sequencing Project, as well as smaller projects such as those described by Akey et al. 2004; Schmid et al. 2005) are not easily analyzed using available software. Here we describe the *mlcoalsim* software application which, unlike other available tools, allows the generation of simulated genetic data and the calculation of descriptive statistics for a large number of loci under different evolutionary models, as well as obtaining *P*-values of observed data.

## Program Overview

*mlcoalsim* enables researchers to compare single and multilocus data with several common evolutionary models. It is an integrated application that not only constructs coalescent trees and sequences but also calculates a number of summary statistics that are useful for the examination of evolutionary hypotheses. This program is designed to generate within-species genetic data; that is, the level of nucleotide variation should not be too high—a maximum of approximately 5%—in order to avoid important errors (a more sophisticated substitution model should be used). For the same reason, the level of divergence from an outgroup species should be no greater than 10–15%.

### Multilocus analyses

One of the main features of *mlcoalsim* is the generation of DNA samples and calculation of a number of statistical tests for a set of multiple loci with variable levels of intragenic recombination. There are two options for multilocus analysis: using independent (unlinked) loci and using a single long region separated into several fragments. The first option (independent loci) allows the independent analysis of each locus and the calculation of summary statistics (the average and variance for all loci of each statistic). This option is useful for contrasting data with demographic models that would affect the entire genome. For such an analysis, a correction factor for population size depending on the chromosomal location of each locus (e.g. autosomal, sexual) is needed. The second option (linked loci) generates samples for an entire linked region and calculates statistics for specified fragments within this region or for a sliding window analysis. The “linked” option is useful in evolutionary processes that affect only specific regions, such as a selective sweep in a recombining region.

### Uncertainty in mutation and recombination rates

Mutation and recombination rates are critical parameters which are usually unknown. In order to consider the uncertainty of these two parameters, *mlcoalsim* can sample the rates from a distribution (uniform and gamma distributions are used) instead of using a fixed value. In addition, *mlcoalsim* can generate samples by fixing the observed values, the number of segregating sites, the minimum number of recombination events and (optionally) the number of haplotypes. This last option is obtained using the rejection method 2 of Tavaré (Tavaré et al. 1997). Posterior distributions for the population mutation and for the population recombination parameter are recorded.

### Heterogeneity in mutation and recombination rate across the sequence

*mlcoalsim* is also able to take into account differences in the mutation and in recombination rates across the studied region. Heterogeneity is modelled with a gamma distribution, modelling from extreme hotspots regions (i.e. in case using heterogeneity for the recombination rate, only few position are enabled to recombine while others can not) to uniform values for all positions. Furthermore, it is possible to fix the average number of invariant positions (position that can not mutate) for the studied region.

### Evolutionary models

*mlcoalsim* includes the following evolutionary models: the neutral stationary panmictic model, the finite island model, models with changing population sizes over time, refugia models and deterministic positive selection (not all of these models can be used simultaneously). *mlcoalsim* allows the use of neutral and positive selection models for different independent loci, and changing population size also can be used with a finite island model.

### Statistics

A number of statistics and related tests used in population genetics are displayed in the output (Table 1). The statistics incorporated in this program describe the level and patterns of diversity for a given sample.

Different statistics that estimate the level of variation are included (θWatterson, 1975, π, Tajima, 1983, and θ*_H_*, Fay and Wu, 2000) for the entire sample. Although these estimates are calculated using different approaches, the values should be equal under the assumption of a neutral stationary panmictic model. The average levels of variation within and among populations are also estimated (π*_w_* and π*_b_*, Hudson et al. 1992), as well as the average differentiation among populations with the *Fst* statistic (e.g. Hudson et al. 1992).

A description of the patterns of diversity is obtained using two main classes of statistics (Ramos-Onsins and Rozas, 2002): Class I statistics, which use the mutation frequency information, and Class II statistics, which use information from the haplotype distribution. Class I includes Tajima’s *D* test (TD, Tajima, 1989), Fu and Li’s tests (FD*, FF*, FD, FF, Fu and Li, 1993), Fay and Wu’s *H* test (Fay and Wu, 2000), *R2* (Ramos-Onsins and Rozas, 2002) and weighted statistics for a multilocus approach such as *D/Dmin* (Schaeffer, 2002) and *H/Hmin* (Schmid et al. 2005). Class II includes the number of haplotypes *Kw* (Strobeck, 1987) and the haplotype diversity *Hw* (Depaulis and Veuille, 1998), both weighted by the number of samples for a better multilocus comparison, *Fs* (Fu, 1997), the statistics *B* and *Q*, (Wall, 1999) which count differences in haplotype structure at adjacent positions, the *ZA* statistic (Rozas et al. 2001) as a measure of linkage disequilibrium at adjacent positions, *maxhap* (Depaulis et al. 2003) and *maxhap1* (simplified from Hudson et al. 1994), which counts the number of lines with the most common haplotype (i.e. *maxhap*) but allowing a single segregating site within the largest “haplotype” group (Rozas et al. 2001). Finally, the minimum number of recombination events, *Rm* (Hudson and Kaplan, 1985), is also calculated.

Multilocus analyses generate a comprehensive output with the calculated statistics with their average and variance. Only biallelic positions are considered for the analyses given that tri- or tetra-allelic positions are rare in within-species samples.

### Other technical features

The generation of random deviates from uniform, binomial, Poisson, and gamma distributions and the determining of roots for complex functions are based on Lanczos (1964); Atkinson (1979); Cheng and Feast (1979); Fishman (1979); Ridders (1979); Press et al (1992) and Press and Teukolsky (1992). The Rm function was obtained and modified from Wall’s code (Wall, 2000). The gamma function was partially obtained from Grassly, Adachi and Rambaut code (Grassly et al. 1997).

## Figures and Tables

**Table 1.** List of the main statistics included in mlcoalsim.

Name	Statistic	Citation
TD	Tajima’s *D* test	Tajima, 1989
Fs	Fu’s *Fs* test	Fu, 1997
FD*	Fu and Li’s D* test	Fu and Li, 1993
FF*	Fu and Li’s F* test	Fu and Li, 1993
FD	Fu and Li’s *D* test	Fu and Li, 1993
FF	Fu and Li’s *F* test	Fu and Li, 1993
H	Fay and Wu’s *H* test	Fay and Wu, 2000
B	Wall’s *B* test	Wall, 1999
Q	Wall’s *Q* test	Wall, 1999
ZA	*ZA*	Rozas et al. 2001
Fst	*F_st_*	Hudson et al. 1992
Kw	No. haplotypes/*n*	Strobeck, 1987
Hw	Haplotype diversity/*n*	Depaulis and Veuille, 1998
R2	*R2* test	Ramos-Onsins and Rozas, 2002
S	No. of biallelic mutations	
thetaWatt	θ	Watterson, 1975
thetaTaj	π	Tajima, 1983
thetaFW	θ*_H_*	Fay and Wu, 2000
pi_w	π within populations	e.g. Hudson et al. 1992
pi_b	π among populations	e.g. Hudson et al. 1992
D/Dmin	*D/Dmin*	Schaeffer, 2002; Schmid et al. 2005
H/Hmin	H/Hmin	Schmid et al. 2005
maxhap	No. lines in most common haplotype/*n*	Depaulis et al. 2003
maxhap1	maxhap excepting one biallelic mutation	Hudson et al 1994; Rozas et al. 2001
Rm	*Rm*	Hudson and Kaplan, 1985

*n* is the number of sequence lines.

See text and *mlcoalsim* documentation for a brief description of statistics.

## References

[b1-ebo-03-41] Akey JM, Eberle MA, Rieder MJ, Carlson CJ, Shriver MD, Nickerson DA, Krugyak L (2004). Population history and natural selection shape patterns of genetic variation in 132 genes. PLoS Biol.

[b2-ebo-03-41] Atkinson AC (1979). The computer generation of poisson random variables. Appl. Statist.

[b3-ebo-03-41] Cheng RC, Feast GM (1979). Some simple gamma variate generators. Appl. Statist.

[b4-ebo-03-41] Depaulis F, Mousset S, Veuille M (2003). Power of neutrality tests to detect bottlenecks and hitchhiking. J. Mol. Evol.

[b5-ebo-03-41] Depaulis F, Veuille M (1998). Neutrality tests based on the distribution of haplotypes under an infinite-site model. Mol. Biol. Evol.

[b6-ebo-03-41] Donnelly P, Tavaré S (1995). Coalescent and genealogical structure under neutrality. Ann. Rev. Genet.

[b7-ebo-03-41] Excoffier L, Novembre J, Schneider S (2000). SIMCOAL: a general coalescent program for the simulation of molecular data in interconnected populations with arbitrary demography. J. Hered.

[b8-ebo-03-41] Fay JC, Wu CI (2000). Hitchhiking under positive Darwinian selection. Genetics.

[b9-ebo-03-41] Fishman GS (1979). Sampling from the binomial distribution on a computer. J. Am. Statist. Ass.

[b10-ebo-03-41] Fu YX (1997). Statistical tests of neutrality of mutations against population growth, hitchhiking and background selection. Genetics.

[b11-ebo-03-41] Fu YX, Li WH (1993). Statistical tests of neutrality of mutations. Genetics.

[b12-ebo-03-41] Grassly NC, Adachi J, Rambaut A (1997). PSeq-Gen: an application for the Monte Carlo simulation of protein sequence evolution along phylogenetic trees. Comput Appl Biosci.

[b13-ebo-03-41] Hudson RR, Futuyama D, Antonovics J (1990). Gene genealogies and the coalescent process. Oxford Surveys in Evolutionnary Biology.

[b14-ebo-03-41] Hudson RR (2002). Generating samples under a Wright-Fisher neutral model of genetic variation. Bioinformatics.

[b15-ebo-03-41] Hudson RR, Bailey K, Skarecky D, Kwiatowsky J, Ayala F (1994). Evidence for positive selection in the superoxide dismutase (*Sod*) region of *Drosophila melanogaster.*. Genetics.

[b16-ebo-03-41] Hudson RR, Kaplan NL (1985). Statistical properties of the number of recombination events in the history of a sample of DNA sequences. Genetics.

[b17-ebo-03-41] Hudson RR, Slatkin M, Maddison WP (1992). Estimation of levels of gene flow from DNA sequence data. Genetics.

[b18-ebo-03-41] Kingman JFC (1982). The coalescent. Stochast. Proc. Appl.

[b19-ebo-03-41] Kingman JFC (1982). On the genealogy of large populations. J. Appl. Prob.

[b20-ebo-03-41] Lanczos C (1964). A precision approximation of the gamma function. J. SIAM.

[b21-ebo-03-41] Laval G, Excoffier L (2004). SIMCOAL 2.0: a program to simulate genomic diversity over large recombining regions in a subdivided population with a complex history. Bioinformatics.

[b22-ebo-03-41] Mailund T, Schierup M, Pedersen CNS, Mechlenborg PJM, Madsen JN, Scauser L (2005). Coasim: A flexible environment for simulating genetic data under coalescent models. BMC Bioinformatics.

[b23-ebo-03-41] Marjoram P, Wall JD (2006). Fast “coalescent” simulation. BMC Genetics.

[b24-ebo-03-41] Nordborg M, Balding D, Bishop M, Cannings C (2001). Coalescent theory. Handbook of Statistical Genetics.

[b25-ebo-03-41] Press WH, Teukolsky SA (1992). Portable random number generators: 6(5):522. Computers in Physics.

[b26-ebo-03-41] Press WH, Teukolsky SA, Vetterling WT, Flannery BP (1992). Numerical Recipes in C The Art of Scientific Computing.

[b27-ebo-03-41] Ramos-Onsins SE, Rozas J (2002). Statistical properties of new neutrality tests against population growth. Mol. Biol. Evol.

[b28-ebo-03-41] Ridders CJF (1979). A new algorithm for computing a single root of a real continuous function. IEEE Transactions on Circuits and Systems.

[b29-ebo-03-41] Rozas J, Gullaud M, Blandin G, Aguade M (2001). DNA variation at the rp49 gene region of Drosophila simulans: evolutionary inferences from an unusual haplotype structure. Genetics.

[b30-ebo-03-41] Schaeffer S (2002). Molecular population genetics of sequence length diversity in the Adh region of Drosophila pseudoobscura. Genet. Res.

[b31-ebo-03-41] Schmid KJ, Ramos-Onsins SE, Ringys-Beckstein H, Weisshar B, Mitchell-Olds T (2005). A multilocus sequence survey in *Arabidopsis thaliana* reveals a genome-wide departure from a neutral model of DNA sequence polymorphism. Genetics.

[b32-ebo-03-41] Spencer CCA, Coop G (2004). *SelSim*: a program to simulate population genetic data with natural selection and recombination. Bioinformatics.

[b33-ebo-03-41] Strobeck C (1987). Average number of nucleotide differences in a sample from a single subpopulation: a test for population subdivision. Genetics.

[b34-ebo-03-41] Tajima F (1983). Evolutionary relationship of DNA sequences in finite populations. Genetics.

[b35-ebo-03-41] Tajima F (1989). Statistical method for testing the neutral mutation hypothesis by DNA polymorphism. Genetics.

[b36-ebo-03-41] Tavaré S, Balding DJ, Griffiths RC, Donnelly P (1997). Inferring coalescence times from DNA sequence data. Genetics.

[b37-ebo-03-41] Wall JD (1999). Recombination and the power of statistical tests of neutrality. Genet. Res.

[b38-ebo-03-41] Wall JD (2000). A comparison of estimators of the population recombination rate. Mol. Biol. Evol.

[b39-ebo-03-41] Watterson G (1975). On the number of segregating sites in genetical models without recombination. Theor. Popul. Biol.

